# Carrot intake and incidence of urothelial cancer: a systematic review and meta-analysis

**DOI:** 10.18632/oncotarget.19832

**Published:** 2017-08-03

**Authors:** Xiao Luo, Hongsun Lu, Yaojun Li, Shijian Wang

**Affiliations:** ^1^ Department of Urology, The First People's Hospital of Tongxiang City, Tongxiang, Zhejiang Province 314500, China

**Keywords:** carrot, urothelial cancer, meta-analysis, risk

## Abstract

Previous studies regarding the relationship between carrot intake and risk of urothelial cancer have reported conflicting results. Hence we performed a meta-analysis of eligible studies to summarize evidence on this association. A comprehensive search up to January 2017 was performed in PubMed, Web of Science, Scopus, EMBASE, Cochrane register, and Chinese National Knowledge Infrastructure (CNKI) databases. The combined odds ratio (OR) with 95% confidence interval (CI) for the highest versus the lowest intake of carrot was calculated. A total of six epidemiological studies consisting of four case-control and two cohort studies were included. Overall analysis indicated a significantly reduced risk of urothelial cancer for high intake of carrot (OR = 0.63, 95% CI 0.44–0.90). Obvious significant heterogeneity was observed among included studies (*P* < 0.001 for heterogeneity; *I*^2^ = 79.6%). There was no significant publication bias by Begg's test (*P* = 0.348) or Egger's test (*P* = 0.130). In conclusion, this meta-analysis indicates that high intake of carrot is associated with a low incidence of urothelial cancer. Considering the limited included studies and huge heterogeneity, further large well-designed prospective cohort studies are warranted to confirm the findings from our meta-analysis.

## INTRODUCTION

Urothelial cancer is the second most common cancer of the genitourinary tract [1]. Urothelial cancer can be located in the lower urinary tract (bladder and urethra) or upper urinary tract (renal pelvis and ureter). Bladder cancer accounts for 90–95% of urothelial cancer and is the most common malignancy of the urinary tract [2]. Although the etiology of urothelial cancer remains largely elusive, tobacco and aromatic amines exposure are generally considered potential risk factors for this cancer [3, 4]. Furthermore, emerging evidence indicates a significant influence of dietary factors, such as fruit and vegetables [5], on urothelial cancer incidence.

In epidemiological studies, such as case-control and prospective cohort studies, the potential relationship between carrot consumption and site-specific cancer incidence has been investigated. A dose-response meta-analysis indicated that carrot intake might be associated with a reduced risk of prostate cancer [6]. Fallahzadeh et al. reported an inverse relationship between the consumption of carrot and gastric cancer risk [7]. Several previous studies also have evaluated the relationship between carrot intake and urothelial cancer [8–13]. However, the findings are not completely consistent, possibly due to lack of sufficient statistical power in the individual studies.

The aim of this study was to evaluate the potential relationship between carrot intake and urothelial cancer risk by performing a meta-analysis of all eligible case-control and cohort studies. We also carried out stratified meta-analysis based on study design, geographical region, gender, and carrot type.

## RESULTS

### Literature search and study characteristics

**Figure 1:**
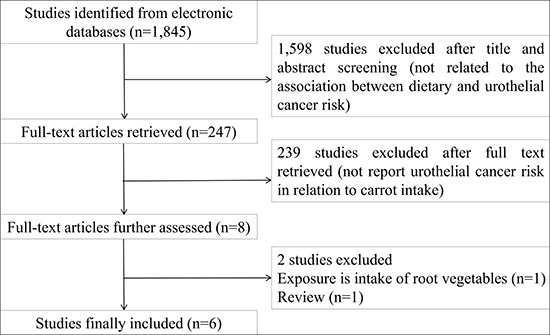
Flow diagram of literature search and study selection

The detailed steps of literature search were present in Figure 1. Six studies [8–13] were included in this meta-analysis on the association of carrot intake with urothelial cancer risk. These studies were performed in the following geographical regions: Europe (*n* = 3), Asia (*n* = 2), and USA (*n* = 1). All included studies were published between 1979 and 2005, of which two were cohort and four were case-control studies. Information on carrot intake was collected by face-to-face interview or a self-administered questionnaire. Table 1 lists the basic characteristics of each study included in our meta-analysis.

**Table 1:** Main characteristics of studies included in this meta-analysis

Author	Year	Country	Design	Number of cases	Total OR (95% CI)	Matched or adjusted variables
Radosavljevic´	2005	Serbia	Case-control	130	0.15 (0.05–0.41)	Age, sex, and residence
Sakauchi	2005	Japan	Cohort	123	1.01 (0.60–1.71)	Age, sex, and smoking
Wakai	2004	Japan	Case-control	124	0.41 (0.16–1.01)	Age, sex, and smoking
Zeegers-1	2001	Netherlands	Case-cohort	538	0.69 (0.51–0.94)	Age, sex, and smoking
Zeegers-2	2001	Netherlands	Case-cohort	538	1.06 (0.97–1.15)	Age, sex, and smoking
Pohlabeln-M	1999	Germany	Case-control	239	0.36 (0.17–0.79)	Age and smoking
Pohlabeln-F	1999	Germany	Case-control	61	2.06 (0.49–8.69)	Age and smoking
Mettlin-M	1979	USA	Case-control	369	0.77 (0.48–1.23)	Age
Mettlin-F	1979	USA	Case-control	110	0.31 (0.13–0.74)	Age

OR, odds ratio; CI, confidence interval.

### Overall and subgroup analyses

**Figure 2:**
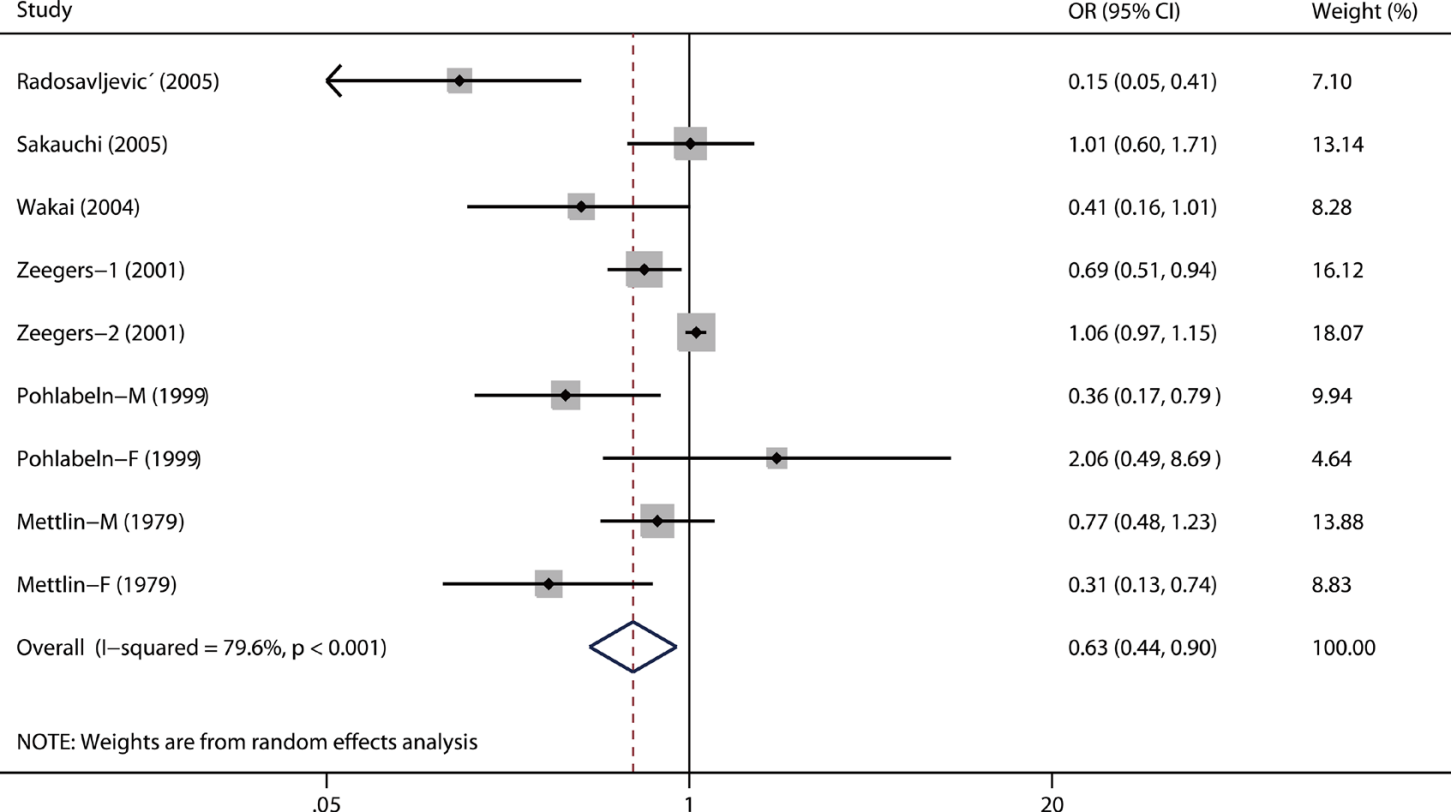
A forest plot showing pooled risk estimate from all eligible studies estimating the association between carrot consumption and risk of urothelial cancer

Figure 2 has plotted the combined odds ratio (OR) with 95% confidence interval (95% CI) for carrot intake. There was a significantly reduced risk of urothelial cancer for high consumption of carrot (OR = 0.63, 95% CI 0.44–0.90). Obvious significant heterogeneity was observed among included studies (*P* < 0.001 for heterogeneity; *I^2^* = 79.6%).

We then conducted subgroup analyses by study design, study region, carrot type, and gender (Table 2). In the subgroup analysis by study design, we found a significantly reduced risk of urothelial cancer in case-control studies (OR = 0.45, 95% CI 0.25–0.81) rather than in cohort studies (OR = 0.91, 95% CI 0.67–1.24). Furthermore, when separately analyzed by carrot type, a more significant association was observed for cooked carrot (OR = 0.69, 95% CI 0.51–0.94) than for raw carrot (OR = 0.84, 95% CI 0.37–1.93). In the stratified analysis by geographical region, the combined ORs (95% CIs) were 0.62 (0.37–1.06), 0.70 (0.29–1.67), and 0.53 (0.22–1.26) for Europe, Asia, and USA, respectively. Finally, the pooled ORs (95% CIs) were 0.56 (0.27–1.17) and 0.73 (0.12–4.60) for male and female, respectively.

**Table 2:** Summary of pooled risk estimates of urothelial cancer in subgroups

Variables	Study number	OR (95% CI)	Heterogeneity assessment		
			Q	P	I^2^ (%)
Total	6	0.63 (0.44–0.90)	39.19	< 0.001	79.6
Carrot type					
Raw	2	0.84 (0.37–1.93)	8.35	0.015	76.1
Cooked	1	0.69 (0.51–0.94)	-	-	-
Study design					
Cohort/case-cohort	2	0.91 (0.67–1.24)	7.03	0.030	71.6
Case-control	4	0.45 (0.25–0.81)	13.95	0.016	64.2
Region					
USA	1	0.53 (0.22–1.26)	3.17	0.075	68.5
Europe	3	0.62 (0.37–1.06)	27.69	< 0.001	85.6
Asia	2	0.70 (0.29–1.67)	2.78	0.095	64.0
Gender					
Male	2	0.56 (0.27–1.17)	2.73	0.099	63.3
Female	2	0.73 (0.12–4.60)	4.83	0.028	79.3

### Influence analysis

**Figure 3:**
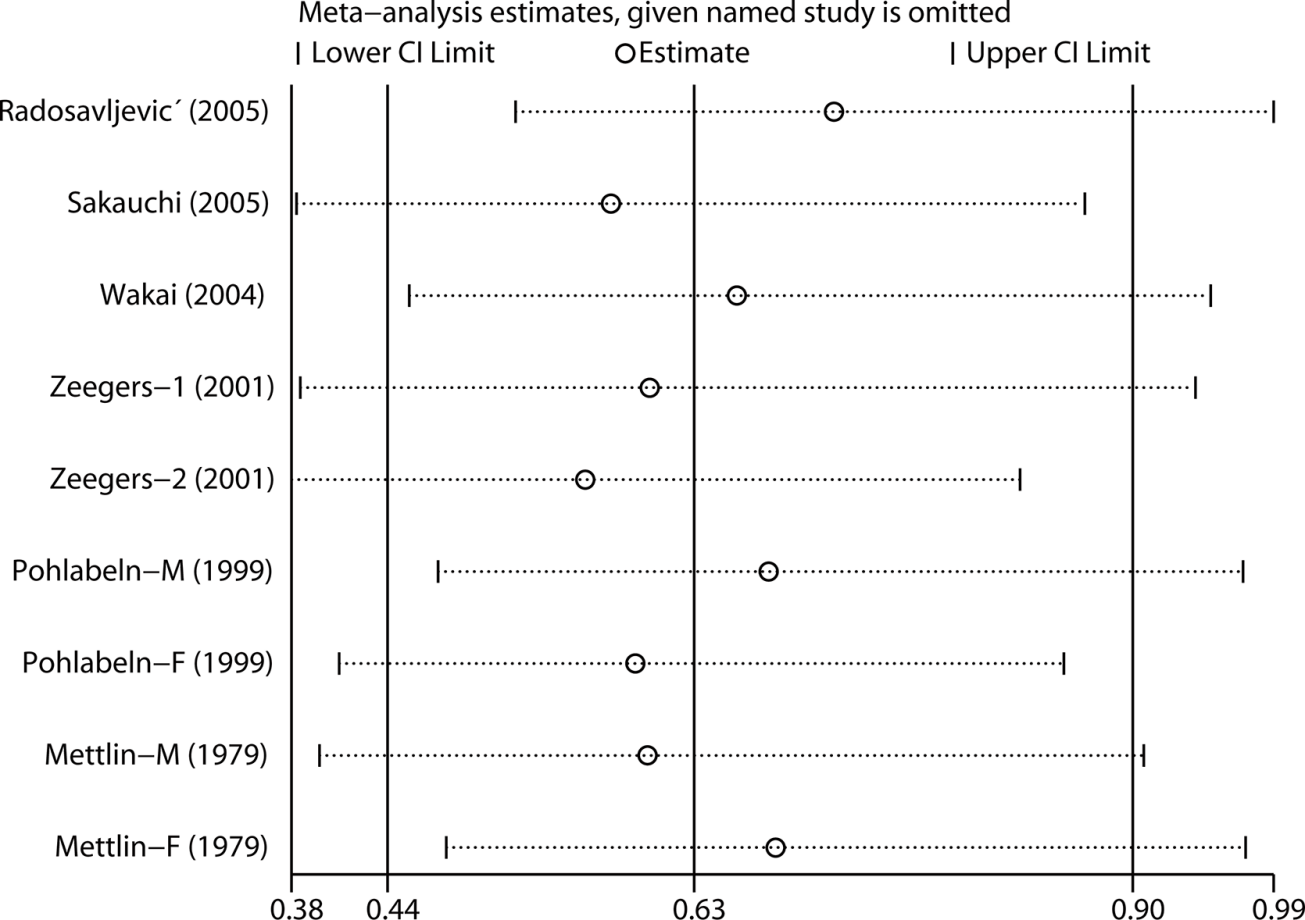
Influence analysis for the effect of carrot consumption on urothelial cancer The analysis was performed by omitting each study in turn.

In the influence analysis, the impact of each study on the combined OR was checked by repeating the meta-analysis after omission of each study in turn. As shown in Figure 3, the combined OR was robust and no single study significantly affected the combined risk estimate.

### Publication bias

There was no significant publication bias by Begg's test (*P* = 0.348) or Egger's test (*P* = 0.130).

## DISCUSSION

The combined risk estimates of this meta-analysis provided limited evidence for a protective association of high carrot intake with urothelial cancer risk. The overall analysis and subgroup analysis in case-control studies indicated a significant reduction in risk, while the result from the cohort studies was null. To the best of our knowledge, this is the first systematic review and meta-analysis aimed to evaluate the relationship between carrot intake and urothelial cancer risk.

In this study, there was statistically significant heterogeneity among included studies (*P* < 0.001). Heterogeneity is often a concern in a systematic review and meta-analysis, due to the variation in study design, sources of study population, sample size, definitions of carrot intake, and so on. Therefore, we performed several stratified meta-analysis based on study design, geographical region, gender, and carrot type. However, obvious heterogeneity was still observed in most subgroups, which indicated that the heterogeneity can't be explained by a single factor.

The biologic mechanism underlining the protective effect of carrot intake in reducing the risk of urothelial cancer is likely to be multifactorial. The protective effect of carrot at least partly is attributed to the high content of carotenoids, which is able to inhibit oxidative damage to DNA at low concentrations and have been hypothesized to be anticancer agents [14]. High total intake of carotenoids was reported to be inversely associated with bladder cancer risk [15]. Specific kinds of carotenoids will be converted into vitamin A, which could exert cytotoxic and cytostatic effects and may reverse the tumor cell to the normal phenotype [16]. Total vitamin A intake, retinol intake, and blood retinol levels were reported to be associated with a lower risk of bladder cancer [17]. Furthermore, Beta-carotene may also contribute to cancer prevention [18].

Several important limitations should be considered in interpreting the results of this meta-analysis. First, the number of eligible studies and total sample size was limited, which might affect the reliability of results. Second, significant heterogeneity was detected in overall analysis and in some subgroup analyses, which might distort the meta-analysis. Third, although Begg's test and Egger's test indicated no publication bias, some inevitable publication bias may exist as small studies and studies with null results are less likely to be published. Fourthly, we failed to perform a dose-response analysis between carrot intake and urothelial cancer risk because of the limited data provided in each included study. Some included studies assessed the “high intake of carrot” and “low intake of carrot” according to the frequency of carrot consumption (e.g., once a week vs. never), while the others used tertiles or grams/day. Furthermore, the species of carrot also varied among included studies. Finally, residual or unknown confounders cannot be completely excluded as a potential bias. Some other special influential lifestyle might also impact the current analysis.

In conclusion, this meta-analysis indicates that high intake of carrot is associated with a low incidence of urothelial cancer. Moderate consumption of carrot has been recommended by many researchers, as carrot intake has been reported to be associated a low incidence of several cancers [6, 7] and protection of vision [19] and cardiovascular system [20, 21]. However, because of the above discussed limitations, further large well-designed prospective cohort studies are warranted to confirm the findings from this meta-analysis.

## MATERIALS AND METHODS

### Study search

We performed a comprehensive search in PubMed, Web of Science, Scopus, EMBASE, Cochrane register, and Chinese National Knowledge Infrastructure (CNKI) databases from their inception to January 2017, using the following search algorithm: (diet or nutrition or vegetable or vegetables or carrot or carrots or carotenoids) and (urothelial cancer or urothelial neoplasm or urinary tract cancer or bladder neoplasm or bladder cancer). We assessed potentially relevant studies by screening their titles and abstracts. Full texts for articles matching the eligible criteria were retrieved. We also examined the cited references from retrieved articles and reviews to identify any additional relevant studies. There was no language limitation. This meta-analysis follows the standards of quality for reporting systematic review and meta-analysis [22].

### Study selection

Studies included in this meta-analysis met all the following criteria: (i) evaluated the relationship between carrot intake and urothelial cancer risk, (ii) had a case-control, nested case-control study, or cohort study design, (iii) reported risk estimate and its 95% CI. If multiple studies from the same general population were available, the largest and most detailed study was included in this meta-analysis [23].

### Data extraction

Data were extracted independently by two reviewers (XL and HL) with a predefined data collection form. Disagreement was resolved by discussing with a third reviewer (YL). For each study, the following information were collected: first author's surname, publication year, the country in which the study was performed, study design, number of cases, type of carrots, mostly adjusted risk estimates for highest versus lowest level of carrot intake, and matched or adjusted confounders. Considering that urothelial cancer is a rare disease, the relative risk (RR) was assumed approximately the same as OR, and the OR was used as the study outcome. Any adjusted ORs with 95% CIs were extracted directly from the original reports or calculated indirectly with the available data.

### Statistical methods

To compute a combined OR with its 95% CI, we extracted the mostly adjusted risk estimates provided in each included study. Homogeneity of ORs across studies was evaluated by Q statistic and the *I^2^* score [24]. The null hypothesis homogeneity was disapproved if the *P* value for heterogeneity was < 0.10 or *I^2^* was > 50%. In this study, the pooled ORs with 95% CIs were estimated with a DerSimonian and Laird random-effects model [25], which takes into account both the within and between-study variances. Subgroup analyses were performed by study design, study region, gender and carrot type. Influence analysis was also performed, in which the meta-analysis was repeated after omission of each study in turn. Potential publication bias was assessed by both Begg's test [26] and Egger's test [27]. All of the above statistical analyses were carried out with STATA 10.0 (StataCorp, College Station, TX, USA), with two-sided *P*-values.
